# Recent Advances in the Application of Piezoelectric Materials in Microrobotic Systems

**DOI:** 10.3390/mi13091422

**Published:** 2022-08-29

**Authors:** Alireza Fath, Tian Xia, Wei Li

**Affiliations:** 1 Department of Mechanical Engineering, University of Vermont, 33 Colchester Ave., Burlington, VT 05405, USA; 2 Department of Electrical and Biomedical Engineering, University of Vermont, 33 Colchester Ave., Burlington, VT 05405, USA

**Keywords:** piezoelectric, microrobotic systems, microelectromechanical systems, sensing, energy harvesting, control systems

## Abstract

Recent advances in precision manufacturing technology and a thorough understanding of the properties of piezoelectric materials have made it possible for researchers to develop innovative microrobotic systems, which draw more attention to the challenges of utilizing microrobots in areas that are inaccessible to ordinary robots. This review paper provides an overview of the recent advances in the application of piezoelectric materials in microrobots. The challenges of microrobots in the direction of autonomy are categorized into four sections: mechanisms, power, sensing, and control. In each section, innovative research ideas are presented to inspire researchers in their prospective microrobot designs according to specific applications. Novel mechanisms for the mobility of piezoelectric microrobots are reviewed and described. Additionally, as the piezoelectric micro-actuators require high-voltage electronics and onboard power supplies, we review ways of energy harvesting technology and lightweight micro-sensing mechanisms that contain piezoelectric devices to provide feedback, facilitating the use of control strategies to achieve the autonomous untethered movement of microrobots.

## 1. Introduction

In recent years, there has been an increasing effort to utilize piezoelectric materials in the development of microrobotic systems. With the growing number of mechanisms that scientists use to develop microrobots, there is a great challenge in designing lighter and more efficient microrobots to achieve a certain level of autonomy. Important reviews on microrobotics have been published with different focuses, such as micro-scale flapping-wing robots [1], biohybrid microrobots [2], light-powered microswimmers [3] and drug delivery microrobots [4], etc. Here, we summarize the recent application of piezoelectric materials for the development of microrobots in the direction of autonomy based on the areas corresponding to its basic challenges, including mechanism, power, sensing, and control.

Piezoelectric materials are widely employed in precision motion due to their distinctive advantages such as quick response, high displacement resolution, high stiffness, high actuating force, and little heat generation [5,6]. These features make piezoelectric materials good candidates for developing the actuating module of microrobots. In 2006, Anton and Sodano [7] reviewed the literature (2003–2006) on power harvesting using piezoelectric materials for self-powered wireless sensor applications, and they updated their review with Safaei [8] to include the literature from 2008 to 2018. Moreover, Mahapatra et al. [9] reviewed the nanostructures of piezoelectric materials, manufacturing methods, and material-specific underpinning concepts. The application of piezoelectric actuators is discussed more specifically in areas such as medical and robotics engineering by Uchino [10]. In 2018, Shevtsov et al. [11] discussed the mathematical modeling, experimental techniques, and computer algorithms for piezoelectric generators. They included the particular effects of piezoceramics, such as the flexoelectric effect, and methods for defect identification. As piezoelectric materials development advanced, computational methods were proposed to contain certain phenomena, including rate-dependent switching in the micromechanical 3D finite element model [12].

The mechanism by which piezoelectric microrobots achieve mobility, as well as the environment in which they are expected to maneuver, are important in the development of microrobots. Each mechanism has specific characteristics suitable for an objective environment. Ambulatory locomotion gives the advantages of mobility on rough surfaces as opposed to the traditional wheeled mechanism [13]. Moreover, increasing the number of legs enables the system to be more robust due to the actuation failure [14]. The inchworm mechanism can gain control of the friction force by exploiting the squeeze film effect [15,16]. To create biologically-inspired flapping-wing microrobots like insects for exploration purposes, high-density actuation power [17] is required, which can be developed using piezoelectric materials. Additionally, amphibious microrobots are designed to conform to the multi-environment [18]. 

The key roles in the operation of the microrobot are the power source to achieve mobility and the way it is transferred to the microrobot. The piezoelectric materials that are used in microrobots require high input voltages, which can reach as high as 220 V [19], creating challenges for power transmission. As a promising strategy, there are a variety of methods based on energy harvesting in the direction of the wireless functionality of microrobots relying on piezoelectric actuation [20]. 

The sensing capabilities involve microrobots or piezo-based devices dealing with their environments to achieve autonomy like their biological counterparts, such as tactile sensing similar to that of nature-inspired insects [21]. Additionally, piezoelectric sensing is investigated in a range of fields such as detecting cracks [22] and human health monitoring [23], which could be used to inspire ideas for microrobotic applications. 

The control strategy is essential for the microrobots to achieve stability and follow trajectories. Due to their lightweight and miniature sizes, microrobots are more sensitive to environmental disturbances. Researchers have attempted to address these challenges by adding dampers [24], taking into account the disturbances [25], and using adaptive, model-free MIMO, nonlinear, and spiking neural network control strategies [26,27,28,29,30]. Moreover, the augmentation of accurate sensors to the system helps enhance the stability control of the microrobot.

In this review, we intend to give an overview of the latest advances in the field of piezoelectric microrobots focusing on their innovation and limiting factors. Therefore, we present the up-to-date applications of piezoelectric mechanisms to the development of microrobots with a focus on power, sensing, and control, so as to recognize the challenges that need more consideration and contribute to the understanding, design, and fabrication of piezoelectric microrobots.

## 2. Movement Mechanism

**Figure 1:**
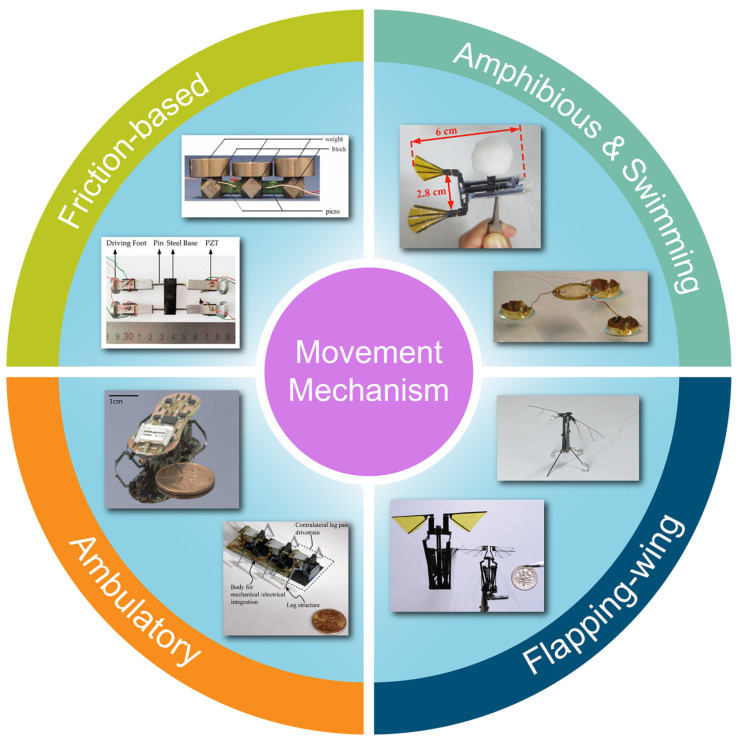
Categories defined for the movement mechanisms of microrobots. Reprinted with permission from Ref. [15]. Copyright 2011, IEEE. Reprinted with permission from Ref. [31]. Copyright 2018, MDPI. Reprinted with permission from Ref. [32]. Copyright 2021, Elsevier. Reprinted with permission from Ref. [18]. Copyright 2013, Springer. Reprinted with permission from Ref. [33]. Copyright 2012, SAGE. Reprinted with permission from Ref. [34]. Copyright 2017, IEEE. Reprinted with permission from Ref. [35]. Copyright 2010, IEEE. Reprinted with permission from Ref. [36]. Copyright 2011, IEEE.

For any specific application, choosing an appropriate type of microrobot is of great importance as they have limited capabilities so far. Several innovative piezoelectric mechanisms have been employed for the movement of microrobots. Here, we divide them into subcategories based on the principles that they use to achieve mobility. Figure 1 depicts the categories defined for microrobots.

### 2.1. Ambulatory Locomotion

**Figure 2:**
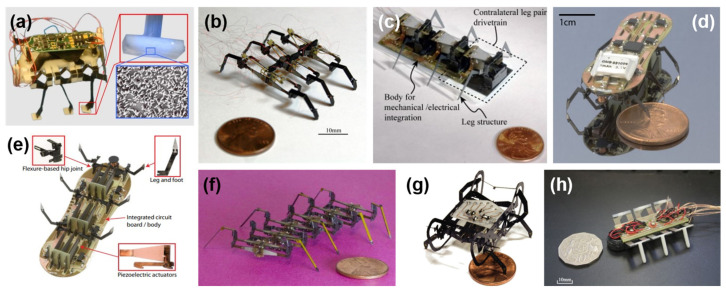
(**a**) Crawler with zoomed-view of fibers in the feet. Reprinted with permission from Ref. [13]. Copyright 2006, IEEE. (**b**) Centipede-like microrobot. Reprinted with permission from Ref. [37]. Copyright 2011, Springer. (**c**) HAMR^2^. Reprinted with permission from Ref. [35]. Copyright 2010, IEEE. (**d**) Top-view and (**e**) bottom-view of HAMR^3^. Reprinted with permission from Ref. [36]. Copyright 2011, IEEE. (**f**) Centipede millirobot. Reprinted with permission from Ref. [14]. Copyright 2013, IEEE. (**g**) HAMR-VP. Reprinted with permission from Ref. [39]. Copyright 2014, SAGE. (**h**) MinRAR. Reprinted with permission from Ref. [40]. Copyright 2017, IEEE.

One of the popular mechanisms is ambulatory locomotion. In 2006, Sahai et al. [13] proposed a 3 g crawling robot that resulted in three prototypes integrating the microtechnologies, as depicted in Figure 2a. Later in 2011, Hoffman and Wood [37] designed and fabricated a myriapod-like microrobot (Figure 2b) equipped with bimorph piezoelectric actuators that weighed 750 mg. This is the improved version of their centipede-inspired microrobot that features better stability. However, it has singularities, and the fabrication process is more complex than the previous version [38]. In 2010, Baisch et al. [35] developed the second generation Harvard Ambulatory MicroRobot (HAMR^2^) that is a biologically-inspired hexapod microrobot (Figure 2c); later in 2011, HAMR^2^ was improved into a lighter autonomous version, which is named HAMR^3^ [36], as shown in Figure 2d,e. Other features such as the robustness of this type of microrobot are discussed by Hoffman and Wood [14], who used redundancy to improve robustness in the case of a leg failure (Figure 2f). In 2014, Baisch et al. proposed the design (Figure 2g) and fabrication of the HAMR-VP [39] to reach a speed as high as biological insects of a similar size, enabling a maximum speed of 10.1 body length per second (440 mm/s). The ambulatory microrobots manufactured by Rios et al. [40] use bimorph piezoelectric benders and can reach a speed of 520 mm/s, as displayed in Figure 2h. Later, Hernando-García et al. [41] increased the maximum speed of such microrobots by comparing the standing and traveling types of wave-based locomotion mechanisms. In comparison, the traveling type of locomotion surpassed the standing type by reaching a terminal velocity of 14 body lengths per second. Moreover, legged microrobots could gain the advantage of a higher payload power ratio by using lead zirconate titanate (PZT)-based actuators [42]. 

Similar microrobots with a focus on the piezoelectric stick-slip motion are mathematically modeled [43], showing that increasing the friction constant results in a lower speed. Additionally, mechanisms were proposed to achieve the stick-slip motion by simultaneously provoking the perpendicular oscillators [44,45]. The ambulatory locomotion-based microrobots are robust to the failure of the individual actuator. However, enabling complex mechanical structures, assembling processes, and onboard electronics pose challenges to their locomotion abilities.

### 2.2. Friction-Based Locomotion

**Figure 3:**
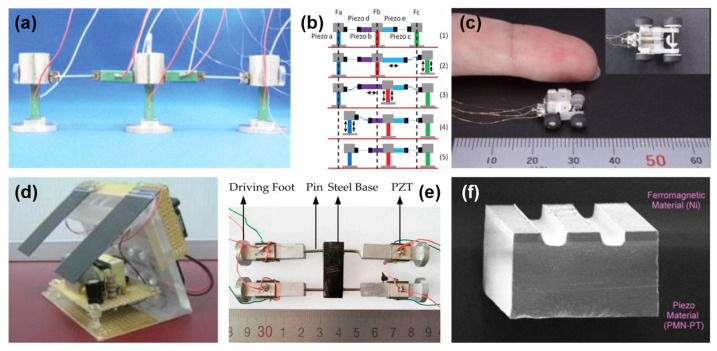
(**a**) Photograph and (**b**) principle of the inchworm microrobot. Reprinted with permission from Ref. [15]. Copyright 2011, IEEE. (**c**) Rolling microrobot. Reprinted with permission from Ref. [46]. Copyright 2021, IEEE. (**d**) Travelling wave-based microrobot. Reprinted with permission from Ref. [47]. Copyright 2010, IEEE. (**e**) Quadruped microrobot. Reprinted with permission from Ref. [31]. Copyright 2018, MDPI. (**f**) MagPier microrobot. Reprinted with permission from Ref. [48]. Copyright 2011, IEEE.

The inchworm microrobot is another type of piezoelectric microrobot that uses friction force to move. Itatsu et al. [15] proposed the use of the squeeze film effect to control the friction force in inchworm microrobots as depicted in Figure 3a. The principle of the inchworm microrobot achieving locomotion is illustrated in Figure 3b. The other ground microrobot that exploits friction to locomote is the rolling type microrobot, as shown in Figure 3c [46]. With a total weight of 640 mg and a velocity of 5.6 mm/s, this microrobot employing micro ultrasonic motors is capable of delivering 60 µNm of torque. Figure 3d shows a type of wireless piezoelectric microrobot [47] that utilizes traveling-wave actuation to move at a speed of 14 mm/s by using 50 V of power supply. Figure 3e demonstrates the quadruped microrobot [31] that uses the bending-bending mode of hybrid oscillation inspired by rowing and can reach a speed of 33.45 mm/s with a weight of 49.8 g. MagPier [48] is a wireless electromagnetic microrobot that uses piezoelectric for sliding actuation (Figure 3f). The friction-based locomotion mechanism benefits from the stability that it has, but the bumpy surface may affect its performance. 

### 2.3. Flapping-Wing Locomotion

**Figure 4:**
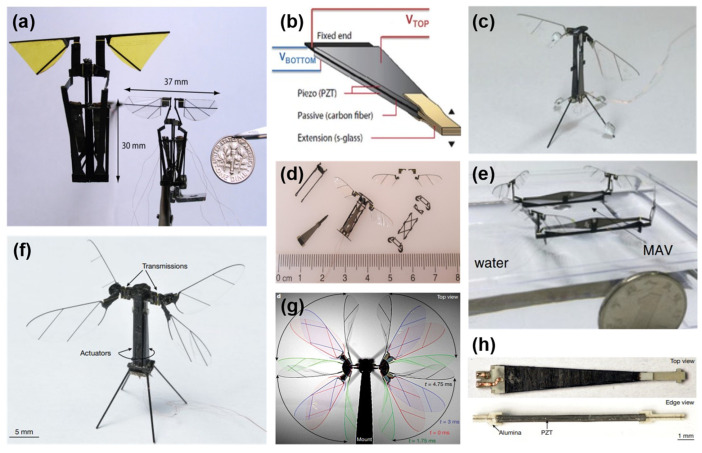
(**a**) Large and small flapping-wing prototypes. Reprinted with permission from Ref. [33]. Copyright 2012, SAGE. (**b**) Bimorph actuator and (**c**) overall structure of RoboBee. Reprinted with permission from Ref. [34]. Copyright 2017, IEEE. (**d**) Whole and separated pieces of flapping-wing microrobot. Reprinted with permission from Ref. [51]. Copyright 2017, Wiley. (**e**) Flapping-wing micro aerial vehicle. Reprinted with permission from Ref. [52]. Copyright 2018, Wiley. RoboBee’s (**f**) X-wing, (**g**) Time-lapse of wing actuation, and (**h**) piezoelectric actuator with alumina and PZT. Reprinted with permission from Ref. [53]. Copyright 2019, Springer.

Another popular mechanism is the flapping-wing microrobot. Durán et al. [49] provided the modeling of piezoelectric actuators necessary for the control of the flapping-wing mechanism that needs high voltage drive electronics [50]. In 2012, Arabagi et al. [33] developed two prototypes of piezoelectric actuators that achieve a 3/8 lift-to-weight ratio (Figure 4a). Ma et al. [17] produced a piezoelectric with high power density for the 80 mg flapping-wing microrobot, capable of flight control, and Lok et al. [34] proposed a low-mass high-voltage drive for piezoelectric actuators with 290 mW power consumption and an overall weight of 70 mg for the RoboBee, which is displayed with its piezoelectric actuator in Figure 4b,c. Furthermore, Zou et al. [51] microfabricated an 84 mg flapping-wing microrobot (Figure 4d) by using a precision assembly of microsystems to achieve high performance. Moreover, a novel design for this concept is shown in Figure 4e, which is a micro aerial vehicle using the flapping-wing integrated within the quadrotor control strategy with a total weight of 247 mg [52]. Jafferis et al. [53] developed an X-wing micro aerial vehicle with lightweight power electronics to accomplish untethered flying via a photovoltaic array, which is shown in Figure 4f with its time-lapse and piezoelectric actuator displayed in Figure 4g,h, respectively. The challenges of achieving high-performance flapping-wing locomotion include developing efficient actuators with a high lift-to-weight ratio and handling the presence of air disturbances that make it difficult to stabilize the flying attitude. Despite these challenges, the advantage of flapping-wing locomotion is obvious: the movement of this type of microrobot is not hindered by ground obstacles.

### 2.4. Amphibious and Swimming Locomotion

**Figure 5:**
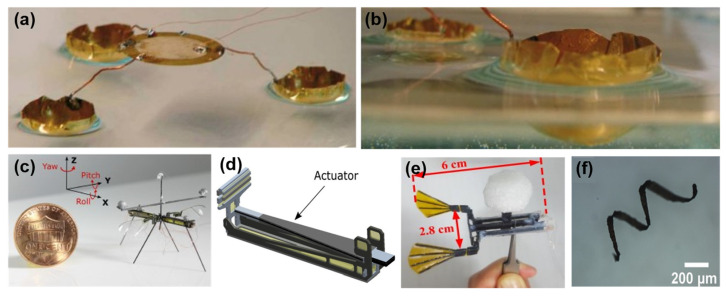
(**a**) Overall structure and (**b**) semi-submerged parts of the amphibious microrobot. Reprinted with permission from Ref. [18]. Copyright 2013, Springer. (**c**) Overall structure and (**d**) actuator of RoboFly. Reprinted with permission from Ref. [54]. Copyright 2021, IEEE. (**e**) Microrobotic fish. Reprinted with permission from Ref. [32]. Copyright 2021, Elsevier. (**f**) Piezoelectric micro swimmer. Reprinted with permission from Ref. [55]. Copyright 2019, Royal Society of Chemistry.

There are other types of movement mechanisms that allow microrobots to operate in different environments. Becker et al. [18] developed a piezoelectric amphibious microrobot capable of locomotion in solid and liquid environments where their prototype, shown in Figure 5a, can reach a speed of 30 mm/s. Its zoomed view of semi-submerged parts is displayed in Figure 5b. Another example is RoboFly [54], which can fly and perform ground and water locomotion movements with a weight of 74 mg, depicted with its actuator in Figure 5c,d. Moreover, RoboFly can land from an unsteady flying position due to its low center of mass compared to its counterparts. There are other types of microrobots operable in liquid environments, such as the micro-robotic fish [32], as shown in Figure 5e. It is powered by bimorph piezoelectric actuators and can reach a speed of 45 mm/s with a weight of 1.93 g. Additionally, for biomedical applications, the magnetic piezoelectric microswimmers [55] have proved to be effective, as they can be powered by magnetic force and steered by piezoelectric polymer (Figure 5f). The amphibious microrobots will gain versatility while being restrained in any individual environment. Although swimming microrobots face the same issues as flying microrobots, they have advantages in various applications, such as biomedical drug delivery and sewer system monitoring. Table 1 gives an overview of the characteristics of the microrobots to provide a clear distinction among different designs.

## 3. Power Supply 

Power source determination and electronics design for piezoelectric microrobots are major challenges in this field. Microrobots usually use wired power supplies at the preliminary stages of innovative mechanism development. However, in this section, we aim to provide a review of the recent technologies that could be used to give enough power for microrobots to move toward wireless autonomy.

In 2008, Karpelson et al. [19] summarized the research on power and actuation for flapping-wing microrobots. In this review, they noted that for sub 1 g micro air vehicles (MAV), a voltage ranging from 110 V to 220 V is needed to drive the piezoelectric bimorph cantilever, which can be exploited to enable 6.75 minutes of maximum flight time with a ten mAh of battery. 

**Figure 6:**
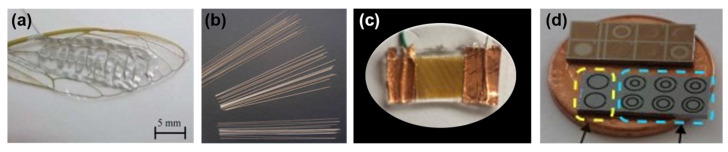
(**a**) Piezoelectric fibers on cicada wings. Reprinted with permission from Ref. [57]. Copyright 2014, Royal Society of Chemistry. (**b**) PZT microfibers. Reprinted with permission from Ref. [58]. Copyright 2012, Elsevier. (**c**) Piezoelectric nanogenerator (PENG). Reprinted with permission from Ref. [59]. Copyright 2019, MDPI. (**d**) Concentric ring boss harvesters (CRBH). Reprinted with permission from Ref. [61]. Copyright 2017, Springer.

Energy harvesting could be another source of power supply for microrobots in the future. In 2011, Kim et al. [20] reviewed the performance and different ways in which piezoelectric materials can harvest energy based on vibrations. Pan et al. [57] used the piezoelectric polypeptide poly(γ-benzyl-L-glutamate) on the wings of the cicada shown in Figure 6a to verify their application and reached a power output of 138.42 pW for one fiber. The use of piezoelectric nanofibers is reviewed in [58], as shown in Figure 6b. You et al. [59] developed aligned P(VDF-TrFE) nanofibers displayed in Figure 6c, being capable of outputting a voltage of 12 V for potential self-powered devices. Moreover, different shapes of piezoelectric energy harvesters have been developed. For example, Kim and Yun proposed a helical piezoelectric capable of stretching up to 158% of its length [60] with a maximum output voltage of 20 V, and Beker et al. demonstrated a circular diaphragm of an aluminum nitride piezoelectric along with a concentric ring boss that can generate 1.3 µW of power (Figure 6d) [61]. Furthermore, there have been theoretical efforts to expand the bandwidth of piezoelectric vibration energy harvesters to absorb more power using nonlinear characteristics [62]. 

Additionally, nanogenerators could be used to harvest energy. In 2016, Li et al. [63,64] introduced the polypropylene ferroelectret to develop a biocompatible, flexible ferroelectret nanogenerator (FENG) that can be used as a dual-functional thin film and can reach a voltage of 50 V by hand pressing [65]. Furthermore, Cao et al. [66] discussed the capability of transferring energy from ferroelectret polymer to energy storage devices and developed a micro-robotic arm controlled by the movement of human fingers through combining the FENG with a VO_2_-based bimorph MEMS actuator [67].

**Figure 7:**
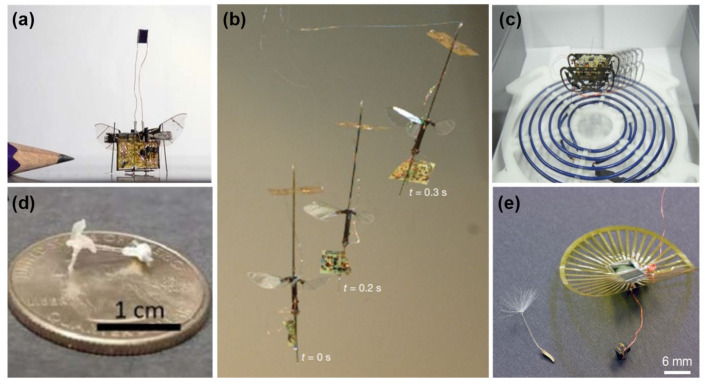
(**a**) Laser-powered insect scale aerial vehicle. Reprinted with permission from Ref. [68]. Copyright 2018, IEEE. (**b**) Untethered flight of flapping-wing MAV. Reprinted with permission from Ref. [53]. Copyright 2019, Springer. (**c**) Wireless-powered HAMR. Reprinted with permission from Ref. [69]. Copyright 2014, IEEE. (**d**) Magnetically driven micro-swimming prototype. Reprinted with permission from Ref. [56]. Copyright 2021, IEEE. (**e**) Wireless wind-dispersed device. Reprinted with permission from Ref. [72]. Copyright 2022, Springer.

For the wireless power transmission, James et al. [68] presented the first laser-powered micro aerial vehicle that needs the power of 200–300 mW and has previously been tethered due to its high voltage, as displayed in Figure 7a. Moreover, Jafferis et al. [53] used three solar light sources to power a 60 mg photovoltaic for an untethered flight of the flapping-wing MAV shown in Figure 7b. In 2021, Bunea et al. [3] reviewed light-powered microswimmers of soft-and hard-responsive types. In addition, the first application of radio frequency (RF) wireless power transfer in microrobots is presented in [69], which can power HAMR with a power requirement of 0.01–1 W (Figure 7c). Figure 7d demonstrates a magnetically actuated micro-swimming robot with 1.5 mT to reach a speed of 19.1 body length per second [56]. Furthermore, Wang and Zhang [70] reviewed the untethered-driven micro-or nanorobots, including their swarm behavior. In addition, there are some microrobots powered by novel methods, such as microswimmers that are powered by microalga [71]; a dandelion-inspired [72] wind-dispersed wireless device that carries solar cells; and a data transmission link that can generate 0.25 mW/mm^2^ (Figure 7e).

## 4. Sensing Capabilities

**Figure 8:**
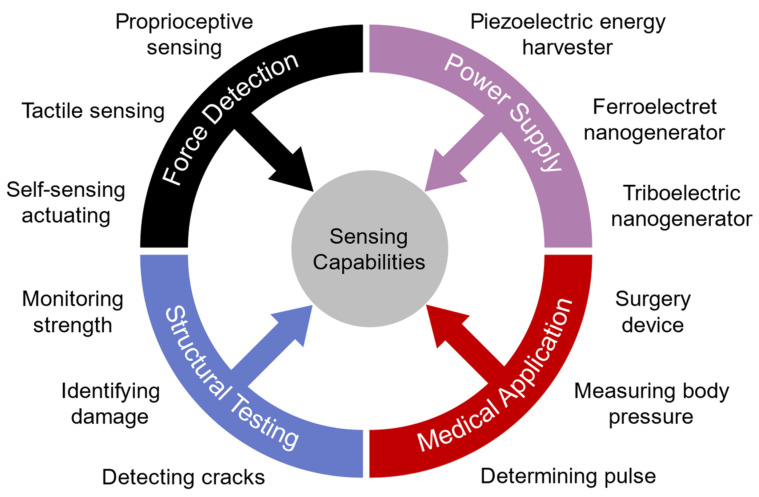
Categories of piezoelectric sensing applications that can be used in microrobots.

As the research interest in microrobotics grows, more sensing capabilities are needed to enable insect-like autonomous robots. In this section, we intend to discuss the research works that utilize piezoelectric devices for sensing in microrobotics and other related fields that could be beneficial for further improving the performance of microrobots. Figure 8 illustrates the classifications of sensing capabilities for microrobotic applications. 

As for the piezoelectric sensors in microrobots, Fahlbusch and Fatikow [73] gave a preliminary overview of force sensing during manipulation in microrobotic systems. Tactile sensors inspired by insects such as spiders and geckos have been investigated by Koç and Akça [21]. In 2014, Lee et al. [74] fabricated a piezoelectric thin-film force sensor that can be used in biomedical applications. Adam et al. [75] developed a microrobot for various environments that has a micro-force sensing magnet. As it is challenging to develop a highly integrated sensing system for microrobots, Jayaram et al. [76] proposed a method to determine the velocity as a function of frequency and voltage in piezoelectric materials. Moreover, proprioceptive sensing in locomotion movement is presented by Doshi et al. [77]. In 2019, Chopra and Gravish [78] used the linear relationship of actuator displacement and voltages with an input of 25 V to 200 V to detect wing collision for flying robots. Furthermore, as vision is an important sensing ability in the environment for insect-sized microrobots, Iyer et al. [79] used a wireless steerable vision on a live beetle that is capable of streaming via Bluetooth radio. Moreover, Han et al. [80] reviewed triboelectric and piezoelectric sensors for displacement, pressure, and acceleration.

Various methods have been used to design piezoelectric sensors. Yamashita et al. [81] developed ultrasonic micro-sensors with PZT thin films and adjusted their resonance frequencies. Chen and Li [82] proposed a PZT-based self-sensing actuator capable of not only generating high-resolution displacement but also monitoring the dynamic characteristics of the mechatronic system. Self-powered piezoelectric materials are studied as both sensors and power generators [83]. In addition, micro-grippers that use micro-force sensors with cantilever structures using piezoelectric biomorphs were investigated [84]. A piezoelectric wireless micro-sensing accelerometer has been studied [85]. In 2017, Hosseini and Yousefi [86] examined the PVDF fabric with the control of crystalline phases for use in flexible force sensors. Hu et al. [87] enhanced piezoelectric sensing by using penetrated electrodes and nanoparticles. Cao et al. [88] used FENG to develop the self-powered bending sensors. 

Moreover, piezoelectric materials can serve as integrated sensors for the structures of robotic systems, such as observing and detecting fatigue cracks [22], as well as identifying structural damage [89]. Moreover, piezoelectric materials have been used as a means of monitoring structural strength [90,91]. In 2010, Feng and Tsai [92] developed a new piezoelectric acoustic emission sensor with PVDF that has a wider bandwidth, and in 2020, Jiao et al. [93] gave a review on structure monitoring with piezoelectric sensing.

**Figure 9:**
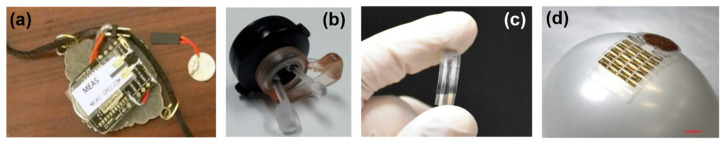
(**a**) Piezoelectric necklace. Reprinted with permission from Ref. [95]. Copyright 2015, Elsevier. (**b**) Pressure-sensing piezoelectric device. Reprinted with permission from Ref. [96]. Copyright 2015, MDPI. (**c**) Biodegradable piezoelectric. Reprinted with permission from Ref. [98]. Copyright 2018, National Academy of Science. (**d**) Installation of the piezoelectric device on a curved balloon. Reprinted with permission from Ref. [23]. Copyright 2019, Wiley.

Additionally, piezoelectric sensors have been used for biomedical applications. Kalange and Gangal [94] utilized piezoelectric sensors to determine human pulse, and Kalantarian et al. [95] used them in a necklace to observe the eating habits, displayed in Figure 9a. Moreover, Figure 9b [96] shows a new device using piezoelectric sensors to measure heart rate by utilizing in-ear pressure data, and Zhou et al. [97] used piezoelectric ceramic to receive signals of sound. In 2018, Curry et al. [98] investigated a piezoelectric biodegradable device (shown in Figure 9c) to measure internal body pressure. As it is essential to have continuous data when sensors are related to vital health monitoring, a wireless self-powered piezoelectric sensor is introduced by Sun et al. (Figure 9d) [23].

There are other applications for piezoelectric sensors, such as using piezoelectric sensors as pressure sensors for obstacle avoidance in underwater vehicles [99], and using PXT/PVDF piezoelectric sensors on the racket for table tennis training purposes [100]. Table 2 provides a summary of the applications of the sensing capabilities of piezoelectric materials.

## 5. Control and Stability

**Figure 10:**
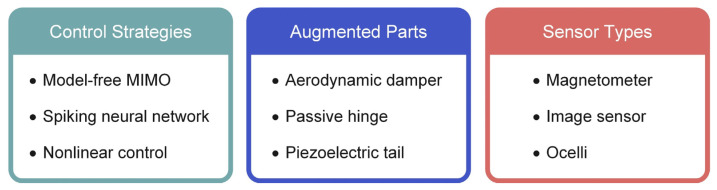
Overview of the technologies used in the control section.

Due to the instability of microrobots, controlling the system is of foremost importance. As flapping-wing microrobots have a higher number of DOFs, controlling them is more challenging. Figure 10 provides a summary of the techniques utilized in this section to achieve the stable control of the system.

**Figure 11:**
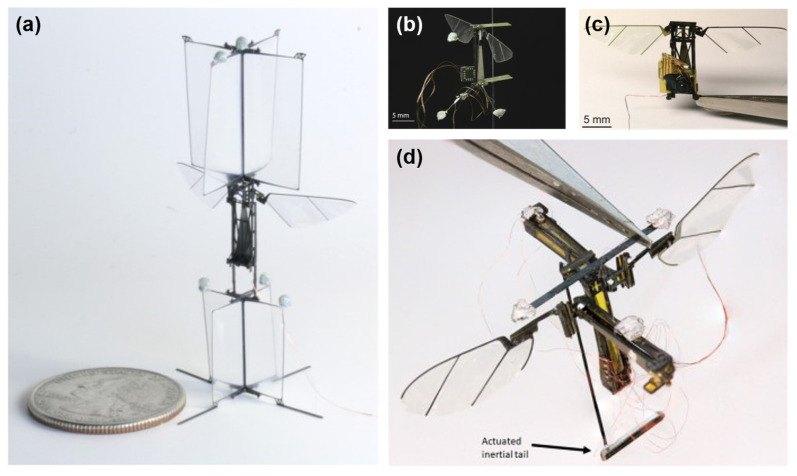
(**a**) RoboBee with aerodynamic damper. Reprinted with permission from Ref. [24]. Copyright 2012, IEEE. (**b**) RoboBee with onboard magnetometer. Reprinted with permission from Ref. [102]. Copyright 2014, IEEE. (**c**) RoboBee with image sensor. Reprinted with permission from Ref. [103]. Copyright 2012, IEEE. (**d**) Flapping-wing microrobot with piezoelectric tail. Reprinted with permission from Ref. [109]. Copyright 2019, IEEE.

Teoh et al. [24] stabilized the flying microrobot by adding an aerodynamic damper and used altitude control on the system by providing feedback with a tracking camera (Figure 11a). Moreover, Teoh and Wood [101] decoupled the control and power to create yaw and roll torques for attitude control by using a spherical four-bar linkage. In an effort to remove external devices for tracking trajectories, Helbling et al. [102] used an onboard magnetometer with a weight of 22 mg for angular feedback to control pitch and yaw (Figure 11b). Duhamel et al. [103] used an image sensor (model: Tam4 chip) to determine the altitude that is biologically inspired for the purpose of making an autonomous microrobot using the LTI system model (Figure 11c). Another type of vision used in flapping-wing microrobots is ocelli, which is inspired by insects and provides angular velocity in the feedback controller [104]. Moreover, different methods are used to control the flight of flapping-wing microrobots, such as adaptive control that is used to track trajectories as a type of lift control strategy [26,29,105]; model-free MIMO control that utilizes experimental methods to detect the behavior of the system, providing key factors for the development of the future model-based controller [27]; and spiking neural network control to mimic the behavior of the insect’s brain and consider the model uncertainties [28]. For enabling yaw control, Teoh and Wood [106] used the fruit fly control strategy that is also capable of creating roll torque by vibrating the hinge, while Chukewad and Fuller [107] utilized three innovative ways, including a novel design, employing wide actuators, and phase shifting on flapping-wing vehicles using a passive hinge. In 2017, Chen et al. [108] simulated the dynamics of flapping-wing microrobots on quadrotors to facilitate the application of control strategies, and Chirarattananon et al. [25] investigated flight control in the case of wind disturbances with prediction and compensation schemes. Moreover, to achieve better control capabilities, the piezoelectric tail has been utilized for fast inertial reorientation [109,110], as displayed in Figure 11d. Chen et al. [111] used soft artificial muscles to achieve open-loop stability and closed-loop flying while proposing solutions to the challenges of using soft actuators. In 2021, James and Fuller [112] provided an electronic high-voltage power source for flying insect microrobots to give the necessary torques and forces for the thrust of the control system.

Control strategies were also investigated on legged insect-scale robots. Ozcan et al. [113] controlled the lateral velocity and orientation of HAMR to follow trajectories and experimented with the maneuverability of the microrobot. Furthermore, Doshi et al. [114] made a model from the experimental data of HAMR and used an off-board phase detector to control the legs at their resonance frequency. Designing lightweight power electronics is a great challenge as well, which is investigated along with onboard straight line and high-speed running control [115].

Nonlinear control methods are effective in microrobots due to their high nonlinearities in the system. Karami et al. [30] designed an ant colony optimization-based optimal nonlinear control method for stick-slip microrobots and noted that a linear optimal PID controller does not provide adequate performance for the system. Additionally, nonlinear control methods have been employed for endovascular microrobots. For example, Arcese et al. [116] satisfied the stability with control Lyapunov functions by using a nonlinear adaptive control law for the magnetic microrobot, and in 2020, Pourmand and Sharifi [117] proposed a nonlinear adaptive sliding mode control method by using the Lyapunov theorem for assuring the stability of the system. In 2022, Jiang et al. [118] reviewed the control of microrobots and suggested that for the nonlinear control of systems with significant uncertainties; robust control methods, for example, $H_{\infty }\;$control and sliding mode control; and for systems without a comprehensive model, nonlinear adaptive control strategies are appropriate. Additionally, Diller et al. [119] presented a method for the control of multiple agents individually using magnetic gradient pulling to follow 3-D trajectories. Yang and Zhang [120] reviewed the motion and control of magnetic microrobots and discussed the challenges of swarm control. 

## 6. Conclusions

In this review, we summarize the recent advances in the application of piezoelectric materials in developing microrobots. A variety of mechanisms are presented with the details of the weight and speed they can reach. The microrobotic field is advancing toward making autonomous untethered microrobots where one challenge is to meet the high voltage requirement for piezoelectric actuators. Here, we review the methods of untethered mobility with power harvesting that would benefit the design of next-generation microrobots. Furthermore, for nature-inspired autonomy, the microrobots require adequate sensing and actuation to formulate a sense of the environment and incorporate control strategies to stabilize the system. Adding a sensor to the microrobot can significantly increase the weight of the robot. Therefore, we conduct reviews of practical approaches along with piezoelectric sensing and control methods to achieve stable orientations and accurate trajectories. As wireless power transmission technologies advance and the sensors become lighter and more accurate, by overcoming the design and fabrication challenges, untethered autonomous piezoelectric microrobots will gain more popularity.

## Figures and Tables

**Table 1:** Summary of microrobots with different movement mechanisms.

Movement Mechanism	Author	Year	Weight	Length Scale	Speed	Ref.
Ambulatory	Sahai et al.	2006	3.1 g	mm	10 mm/s	[13]
Ambulatory	Hoffman and Wood	2011	750 mg	mm	0.3 mm/s (0.1 bl/s)	[37]
Ambulatory	Baisch et al.	2014	1.27 g	mm	440 mm/s (10.1 bl/s )	[39]
Ambulatory	Rios et al.	2017	16 g	mm	520 mm/s	[40]
Ambulatory	García et al.	2021	250 mg	mm	280 mm/s (14 bl/s)	[41]
Friction-based	Hutama et al.	2021	640 mg	mm	5.6 mm/s	[46]
Friction-based	Pan et al.	2010	100 g	mm	14 mm/s	[47]
Friction-based	Su et al.	2018	49.8 g	mm	33.45 mm/s	[31]
Flapping-wing	Ma et al.	2013	80 mg	mm	-	[17]
Flapping-wing	Lok et al.	2017	70 mg	mm	-	[34]
Flapping-wing	Zou et al.	2017	84 mg	mm	-	[51]
Flapping-wing	Zhou et al.	2018	247 mg	mm	-	[52]
Amphibious	Becker et al.	2013	2.5 g	mm	30 mm/s	[18]
Amphibious	Chukewad et al.	2021	74 mg	mm	5 mm/s	[54]
Swimming	Zhao et al.	2021	1.93 g	mm	45 mm/s	[32]
Swimming	Sui et al.	2021	-	mm	19.1 bl/s	[56]

bl/s—body lengths/second.

**Table 2:** Application of sensing capabilities of piezoelectric materials.

Authors	Application	Ref.
Fahlbusch and Fatikow	Force sensor in microgripper	[73]
Koç and Akça	Tactile sensing	[21]
Lee et al.	Biomedical applications	[74]
Adam et al.	Real-time micro-force sensing	[75]
Jayaram et al.	Control and tracking trajectories	[76]
Doshi et al.	Leg trajectories estimation and control	[77]
Chopra and Gravish	Detecting wing-collision	[78]
Iyer et al.	Object tracking	[79]
Yamashita et al.	Measurement of position	[81]
Chen and Li	Monitoring displacement and dynamic features	[82]
Ng and Liao	Self-powered sensors	[83]
Huang et al.	Identifying micro-force	[84]
Shen et al.	Measuring acceleration	[85]
Hosseini and Yousefi	Flexible force sensor	[86]
Hu et al.	Dynamic loading observation	[87]
Cao et al.	Athletic performance	[88]
Ihn and Chang	Identifying fatigue cracks	[22]
Xu et al.	Structural damage identifying	[89]
Shin et al.	Structural strength monitoring	[90]
Chen et al.	Structural strength monitoring	[91]
Feng and Tsai	Industrial transducers	[92]
Kalange and Gangal	Human pulse measuring	[94]
Kalantarian et al.	Monitoring eating habits	[95]
Park et al.	Heart rate measurement	[96]
Zhou et al.	Sound signal detection	[97]
Curry et al.	Internal body pressure	[98]
Sun et al.	Continuous health monitoring	[23]
Asadnia et al.	Avoiding obstacles	[99]
Tian et al.	Training for table tennis	[100]

## Data Availability

Not applicable.
